# Shared mechanisms: osteoporosis and Alzheimer’s disease?

**DOI:** 10.18632/aging.101828

**Published:** 2019-02-19

**Authors:** Christine M. Dengler-Crish, Florent Elefteriou

**Affiliations:** 1 Department of Pharmaceutical Sciences, Northeast Ohio Medical University, Rootstown, OH 44272, USA; 2 Department of Orthopedics, Baylor College of Medicine, Houston, TX 77030, USA; 3 Department of Molecular and Human Genetics, Baylor College of Medicine, Houston, TX 77030, USA

**Keywords:** osteoporosis, Wnt, serotonin, dementia, Alzheimer’s disease

Bone loss and Alzheimer’s disease make an unexpected, but increasingly common combination in the aging population. The vastly different clinical presentations of these conditions made it hard to envision that a complex brain disease known for destroying our most advanced cognitive abilities could also impact the fundamental framework of the human body. This bias has likely contributed to the dearth of investigation into mechanisms of bone loss in Alzheimer’s disease (AD)-- which presents as a very real and unique problem for these patients.

Osteoporosis and bone fracture are estimated to occur in AD patients at over twice the rate as similarly-aged neurotypical adults [1]. Occurring across international demographics and in both sexes, skeletal problems in AD patients are not a coincidence of aging, nor are they the result of disease-related immobility, as they often precede AD diagnosis [2]. In fact, studies have used BMD to stratify neurotypical subjects 65 years and older into groups at greatest risk for developing dementia—with those exhibiting the lowest bone densities most likely to receive an AD diagnosis within 5-10 years [3]. This is not to say that every adult with osteoporosis will develop AD, however, the fact that over half of AD patients do experience bone deficits merits further consideration as skeletal fracture can detrimentally impact quality of life and diminish life expectancy of this already-vulnerable population.

The little empirical evidence that does exist on this subject makes a compelling case that the neuropathophysiological features of AD may also drive bone loss. To date, three genetic mouse models of AD (i.e. APPswe, APP/PS1ΔE9, and htau mice) have been characterized with a “pre-clinical” low BMD; however, there are possibly many more among the 150+ available AD models that have not been investigated for bone loss. What has been found among these three models is a low bone mass phenotype at ages just preceding the onset of significant hallmark brain pathology and detectable across models representing each hallmark pathology of AD: amyloid beta (Aβ) and phosphorylated tau (ptau)—with data implicating separate mechanisms by which each pathology disrupts skeletal homeostasis.

Data from Aβ dominant APPswe and APP/PS1ΔE9 models support a bone-cell-autonomous role for Aβ in damaging bone tissue, with evidence that Aβ interfaced directly with bone cells to enhance the bone-resorbing activity of osteoclasts and inhibit the bone-building function of osteoblasts [4]. Data obtained from studies with htau mice which selectively develop ptau and neurofibrillary tangle brain pathology illustrated a different mechanism of skeletal disruption. These investigations born out the Dengler-Crish laboratory showed that ptau—which is largely relegated to the neuronal cytoskeleton, damages the serotonergic dorsal raphe nucleus (DRN) of the brainstem in young htau mice [5]. Serotonergic inputs from the DRN to the hypothalamus form a circuit that is essential for maintaining healthy bone mass in adult mammals; hence these findings suggest this circuit is compromised by ptau pathology in htau mice.

Collectively, this research supports unique roles for both Aβ and ptau pathology as contributors to bone loss in AD, but falls short of providing a unifying explanation for bone loss in AD that could apply to either pathological protein--given that both occur in AD but emerge at different times. In light of this, the Dengler-Crish laboratory recently uncovered a potential unifying mechanism that could account for Aβ and ptau pathogenesis as well as bone loss in AD: deficits in Wnt/β-catenin signaling. Wnt/β-catenin signaling is known to facilitate bone formation in bone tissue and to promote synaptogenesis in the brain. Likewise, pathological inhibition of this pathway has been implicated in both osteoporosis and AD pathogenesis, albeit in separate contexts. In disease, suppression of the Wnt pathway allows the production of ptau and Aβ to go unchecked—causing these pathological proteins to accumulate and in turn, activate inflammatory pathways that further inhibit Wnt signaling [6]. This creates a dangerous feed-forward cycle of AD pathogenesis and Wnt deficit. The Dengler-Crish laboratory recently showed evidence that Wnt/β-catenin signaling was disrupted in both bone and brain of htau mice [7]. Importantly, data showed that this Wnt deficiency was detectable in htau bone first—at the earliest age bone loss was apparent, but before Wnt deficits were present in htau brain (unpublished data). One could hypothesize that initial impairments in Wnt signaling occur outside the brain in AD, driving early bone loss but also promoting peripheral accumulation of Aβ that intensifies Wnt inhibition throughout multiple tissues and then ultimately to the brain, where it facilitates ptau and Aβ pathogenesis that leads to eventual neurodegeneration and alteration of the serotonergic pathway controlling bone remodeling. In this scenario, early bone loss would serve as the metaphorical “canary in a coal-mine,” indicating a systemic deficiency in Wnt signaling that will eventually intensify and facilitate brain pathology in AD. As such, more empirical studies are needed to substantiate these ideas.

If such a premise holds true, it would open up a new opportunity for early intervention in AD that involves Wnt signaling restoration. However, this avenue is not without complications. Over-activation of canonical Wnt signaling is the catalyst for many cancers. Therefore, Wnt-activating treatments would need to be targeted to systems where signaling is known to be deficient and titrated to restore levels rather than broadly enhance them. Adding further complication, there is a paucity of selective Wnt agonists available, given the decades of cancer drug development that sought to antagonize this system. Despite these challenges, the Wnt pathway remains an intriguing topic in the search for mechanisms of bone loss in AD—work that may provide warning signs for incipient disease, address quality of life issues in patients with dementia, and identify new mechanisms for slowing AD’s progression.

## References

[1] Weller IM. Ann Epidemiol. 2000; 10:461. 11018377 10.1016/s1047-2797(00)00085-5

[2] Birge SJ, et al. Clin Geriatr Med. 1994; 10:589–609. 10.1016/S0749-0690(18)30318-5 7850692

[3] Zhou R, et al. Curr Alzheimer Res. 2014; 11:706–13. 10.2174/1567205011666140812115818 25115539

[4] Xia WF, et al. J Bone Miner Res. 2013; 28:2122–35. 10.1002/jbmr.1954 23649480 PMC7104794

[5] Dengler-Crish CM, et al.. J Alzheimers Dis. 2017; 55:1605–19 10.3233/JAD-160658 27814296 PMC5181667

[6] Inestrosa NC, Varela-Nallar L. J Mol Cell Biol. 2014; 6:64–74. 10.1093/jmcb/mjt051 24549157

[7] Dengler-Crish CM, et al. Neurobiol Aging. 2018; 67:148–58. 10.1016/j.neurobiolaging.2018.03.021 29660685

